# Revisiting the Effect of Acute *P. falciparum* Malaria on Epstein-Barr Virus: Host Balance in the Setting of Reduced Malaria Endemicity

**DOI:** 10.1371/journal.pone.0031142

**Published:** 2012-02-08

**Authors:** Shamanthi Jayasooriya, Andrew Hislop, Yanchun Peng, Debbie Croom-carter, Ya Jankey, Andrew Bell, Tao Dong, Sarah Rowland-Jones, Alan Rickinson, Michael Walther, Hilton Whittle

**Affiliations:** 1 Medical Research Council Laboratories, Fajara, The Gambia; 2 School of Cancer Studies, Vincent Drive, University of Birmingham, Birmingham, United Kingdom; 3 The Weatherall Institute of Molecular Medicine, University of Oxford, Oxford, United Kingdom; 4 Laboratory of Malaria Immunology and Vaccinology, National Institute of Allergy and Infectious Diseases, National Institutes of Health, Rockville, Maryland, United States of America; 5 Faculty of Infectious and Tropical Diseases, London School of Hygiene and Tropical Medicine, London, United Kingdom; University of Sussex, United Kingdom

## Abstract

Burkitt's lymphoma (BL), an EBV-associated tumour, occurs at high incidence in populations where malaria is holoendemic. Previous studies in one such population suggested that acute *P.falciparum* infection impairs EBV-specific T-cell surveillance, allowing expansion of EBV infected B-cells from which BL derives. We re-examined the situation in the same area, The Gambia, after a reduction in malaria endemicity. Cellular immune responses to EBV were measured in children with uncomplicated malaria before (day 0) and after treatment (day 28), comparing EBV genome loads in blood and EBV-specific CD8^+^ T-cell numbers (assayed by MHC Class I tetramers and IFNγ ELISPOTS) with those seen in age- and sex-matched healthy controls. No significant changes were seen in EBV genome loads, percentage of EBV-specific CD8^+^ T-cells and IFNγ producing T-cells in acute versus convalescent samples, nor any difference versus controls. Regression assays performed also no longer detected any impairment of EBV-specific T-cell surveillance. Acute uncomplicated malaria infection no longer alters EBV-specific immune responses in children in The Gambia. Given the recent decline in malaria incidence in that country, we hypothesise that gross disturbance of the EBV-host balance may be a specific effect of acute malaria only in children with a history of chronic/recurrent malaria challenge.

## Introduction

EBV is a gamma herpesvirus, ubiquitous in human populations, that is usually acquired early in life by the oral route, replicates locally through a virus productive (lytic) infection at oropharyngeal sites and is carried thereafter for life as a latent, largely asymptomatic, infection of the B-cell pool [1], [2]. Yet this apparently innocuous agent has potent growth-transforming ability for its principal target cell, the B lymphocyte, and is linked to several B-cell malignancies especially in immunosuppressed hosts [3].

Indeed EBV first colonises the B-cell system through a growth-transforming infection, where coordinate expression of specific latent cycle genes drives the expansion of infected B-cell clones. These expansions are controlled by the EBV-specific T-cell response but it appears that some infected cells survive by extinguishing latent gene expression, thereby generating a reservoir of latently infected, resting B-cells through which the virus persists [1]. Reactivations from this reservoir do nevertheless occur and, if not controlled by virus-specific T-cells, can lead to further B-cell transformation events and greatly increased loads of latently-infected B-cells in the blood [4]. In the West, this is most apparent in heavily T-cell suppressed transplant patients, where high circulating EBV loads often precede the appearance of EBV-positive post-transplant lymphoproliferative disease (PTLD) [5], [6]. A somewhat analogous situation is thought to occur in Africa, where the similar geographic distributions of Burkitt Lymphoma (BL), an EBV-associated childhood tumour, and of holoendemic malaria suggests that malaria may predispose to BL through suppression of T-cell surveillance leading to increased viral loads [7].

Several studies have indeed implied that malaria exposure can cause a reduction in EBV-specific T-cell responses [8]. Early assays quantifying the regression of EBV-infected B-cell outgrowth in peripheral blood mononuclear cell cultures, an effect mediated by in vitro reactivation of EBV latent antigen-specific CD8^+^ cytotoxic T-cells [9], showed a slight relaxation of T-cell control in healthy adults from holoendemic malarial versus non-endemic malarial areas of New Guinea [10]. Subsequently a study of healthy children in Kenya with differential malaria exposure has shown that a smaller proportion of children, five to nine years of age, had EBV-specific IFN-γ ELISPOT responses (PBMC), when living in malaria-holoendemic areas, compared to those living in areas where exposure is only sporadic [11]. EBV genome loads in whole blood of these children were also significantly higher in the holoendemic malaria group, but only in children one to four years of age [12]. Turning to acute malaria itself, T-cell control as measured by the regression assay appeared to be completely abrogated in children from a holoendemic area, The Gambia, during an episode of acute malaria, but recovered to some extent after treatment and the resolution of symptoms [8], [13]. More recently EBV-specific CD8^+^ T-cell responses restricted through two HLA alleles, B*3501 and B*5301, were measured by IFN-γ ELISPOT in Gambian children with acute malaria and compared to results obtained up to 6 weeks post-treatment. With the caveat of a small sample size in mind, this comparison suggested that responses were impaired during acute disease, but it was not clear whether this reflected a reduction in the numbers or in the functional competence of EBV specific T-cells in the circulation [14].

A further heterogeneous group of studies have looked at EBV viral load dynamics during malaria exposure [15], [16], [17]. Comparability of these results is difficult, as the assays have been conducted on differing sample types, either whole blood, plasma alone or PBMC preparations. Yone et al. saw significantly higher whole blood viral loads in acute uncomplicated malaria cases compared to convalescence, with a similar non-significant trend for complicated malaria cases [17]. In the Gambia, PBMC EBV genome loads did not differ between acute and convalescent malaria cases, but were elevated in the cases compared to healthy controls [14].

Interestingly, the intensity of malarial infection in the Gambian population has reduced substantially [18], [19] since the conduct of those earlier studies demonstrating impaired T-cell control of EBV infection during acute malarial episodes [8], [14]. We therefore set out to re-examine these various parameters of EBV load and EBV-specific CD8^+^ T-cell immunity in these new circumstances. To do so, we assayed EBV-specific CD8^+^ T-cell responses using the two functional assays (regression and IFN-γ ELISPOT) that had been used separately in earlier work [8], [14], and for the first time also enumerated the numbers of EBV epitope-specific cells by HLA class I-peptide tetramer staining. Assays were conducted during acute and convalescent stages of uncomplicated *P. falciparum* infection in children aged 1–15 yrs and in healthy age- and sex- matched control children without malaria.

## Results

All malaria cases were both slide and PCR positive with a geometric mean slide parasite density of 135,878/ul (95% CI 91,194–202,456). All controls were slide and PCR negative for *P. falciparum* parasites. All of the malaria cases and all except one of the controls who was excluded from the analyses were EBV positive by IgG VCA ELISA. Consistent with previous findings [12], [14] there was a significant inverse correlation between EBV genome load and age, Rho = −0.3587, p = 0.0440 (data not shown). 84% of the children had at least one of the HLA restrictions of interest (A2, B35, B8, B53).

### Haematological indices, Malaria exposure and Cellular frequencies in *P. falciparum* infected and uninfected children

Table 1 shows that the two groups of infected and uninfected children were similar with regard to age and sex, indicating successful matching. As expected there was a significant decline in haemoglobin level during the acute phase of malaria, which on convalescence is not significantly different to that found in uninfected controls.

**Table 1:** Age, sex and haemoglobin levels of uncomplicated *P.falciparum* cases and aparasitaemic controls.

	Acute *p.falciparum*	Convalescent *p,falciparum*	Aparasiteamic Controls	Acute versus Convalescent^1^	Convalescent versus Controls^2^
Age (yrs)	7 (4.8–10.0)	-	7 (4.5–9.5)	-	0.95
Male∶Female Ratio	1.91	-	2.00	-	-
Haemoglobin (g/dl)	11.25 (9.83–12.63)	12.50 (11.40–12.93)	12.40 (11.65–13.20)	0.0038	0.65

For age and haemoglobin levels the median value and inter-quartile range are shown in brackets.

1 p value calculated using Wilcoxon rank statistical comparison of matched pairs.

2 p value calculated using Mann-Whitney U statistical comparison of unpaired data.

**Figure 1:**
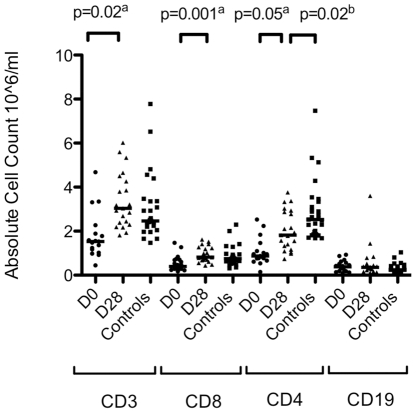
Cellular frequencies in *P. falciparum* infected and uninfected children. There is a significant decrease in the numbers of CD3, CD8 and CD4 positive cells in acute uncomplicated *P. falciparum* malaria (D0, circles), compared to cell numbers during convalescence in the same donors (D28, triangles). The number of CD4 positive cells on convalescence remains significantly lower than age- and sex- matched healthy controls (squares). The number of CD19 positive B-cells doesn't significantly differ. **^a^** *P* value calculated using Wilcoxon rank statistical comparison of matched pairs. **^b^** *P* value calculated using the Mann-Whitney U comparison of unpaired data.

Absolute CD4^+^ and CD8^+^ T-cell counts were significantly lower during acute (Day 0) *P. falciparum* infection compared to convalescence (Day 28) consistent with previous findings [20], [21]. The total number of B-cells however did not significantly differ between acute and convalescent samples or between infected (Day 0 or Day 28) and uninfected control children (Figure 1).

### EBV genome loads and EBV-specific CD8^+^ T-cell responses did not significantly differ between *P. falciparum* infected and uninfected children

**Figure 2:**
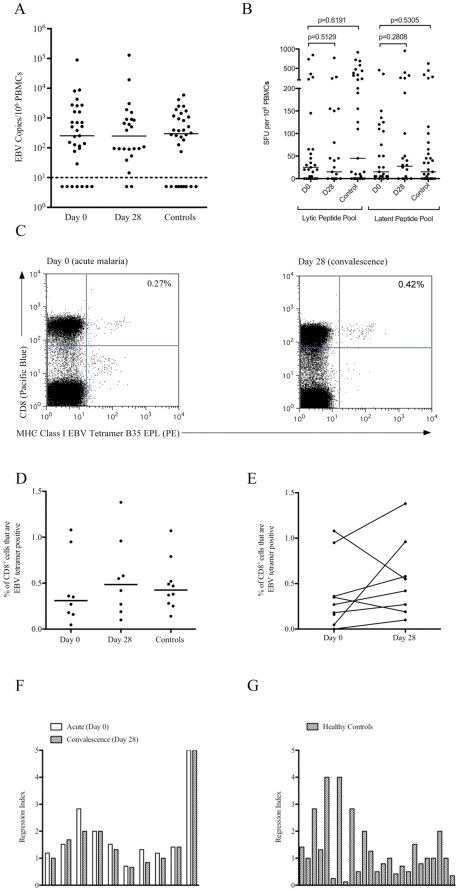
EBV genome loads and EBV-specific CD8^+^ T-cell responses did not significantly differ between P. falciparum infected and uninfected children. A, Epstein-Barr virus (EBV) genome loads during acute uncomplicated P. *falciparum* malaria infection (n = 31), on convalescence in the same donors (n = 24), and from healthy age- and sex- matched controls (n = 32). The median values, illustrated by the solid black lines, for the groups were 253, 249 and 296 EBV copies/1×10^6^ peripheral blood mononuclear cells respectively. The dashed line represents the lower limit of detection for EBV genomes in the assay. Donors below the dashed line had undetectable viral loads. B, Interferon-gamma (γ)-enzyme linked immunospot assays measured the responses to pools of lytic and latent EBV peptides in 27 donors with acute uncomplicated *P. falciparum* infection (Day 0), on convalescence in 23 of the same donors (Day 28) and in 32 age- and sex-matched healthy controls. The solid black lines indicate the median responses for each group. C, A flow cytometry plot from a representative donor (ID740) displaying MHC Class I Tetramer (B35 EPL) against CD8^+^ T-cells. D, The percentage of EBV MHC Class I Tetramer (B*35/EPL, A*2/GLC, A*2/CLG or B*8/RAK) positive CD8^+^ T-cells in acute uncomplicated *P. falciparum* infection (n = 8), on convalescence in the same donors (n = 8) and in age- and sex- matched healthy controls (n = 10). E, The kinetics of the paired MHC class I tetramer responses. F, Regression assays performed on cryopreserved peripheral blood mononuclear cells collected during acute (open bars) and convalescent phases (filled bars) of uncomplicated and complicated *P. falciparum* malaria infection. The last two donors are EBV seronegative controls. The median values in the acute and convalescent groups were 1.47 and 1.37 respectively (*P* = 0.60). G, Ex vivo Regression assays performed on healthy control children.

In contrast to previous findings [14] we found no difference in EBV genome loads between children with acute (Day 0) *P. falciparum* malaria and age- and sex- matched healthy controls (p = 0.749). Likewise, the EBV genome loads did not change significantly between D0 and D28 (p = 0.2173) (Figure 2A). Considering the number of B-cells remained constant (Figure 1) it is unlikely that an increase in the total number of B-cells would have obscured an increase in total EBV genome load. There were 7, 2, and 9 donors with undetectable viral loads in acute (D0), convalescent (D28) and control groups respectively, although all had a positive EBV serology.

Of the 84% of donors with appropriate HLA types for the lytic peptide pool there were 7 (29%), 7 (30%) and 8 (31%) non-responders in the acute (Day 0), convalescent (Day 28) and control groups, respectively. Numbers of non-responders were of a similar magnitude for the latent peptide pool, being 6 (25%), 9 (39%) and 8 (31%) for acute (Day 0), convalescent (Day 28) and control groups, respectively. Background values were of a similar magnitude in all groups, with medians of 15 SFU/10^6^ PBMCs in Day 0, 28 and control groups. Consistent with the observations made for viral loads EBV-specific IFN-γ ELISPOT counts didn't differ between children with acute (Day 0) *P. falciparum* malaria and age- and sex- matched healthy controls to both lytic (p = 0.096) and latent (p = 0.3738) peptide pools. Likewise, there was no significant difference between Day 0 and Day 28 to both lytic (p = 0.9374) and latent (p = 0.0998) peptide pools. (Figure 2B). The results did not alter when all non-responders were excluded from the analysis, when analysed without deduction of the background values and when analysed taking into account the decline in CD8% between Day 0 and Day 28 (Data not shown). Analysis of these data looking at subgroups of different age ranges (0–7 yrs and 8–15 yrs) also showed no significant differences in viral loads and IFN-γ ELISPOTs between Day 0 and Day 28 (Data not shown). Figures 2C–E show that there was no significant difference between the percentages of EBV-specific MHC Class I tetramer positive cells during acute (Day 0) infection and age- and sex- matched healthy controls (p = 0.3599), consistent with our ELISPOT findings. No significant difference was seen between Day 0 and Day 28 (p = 0.4688).

To corroborate these findings we carried out regression assays on cryopreserved PBMCs from a subset of acute uncomplicated and severe *P. falciparum* infected children (n = 9) during the following malaria season. Consistent with our current EBV genome, IFN-γ ELISPOT and MHC Class I Tetramer data, but in contrast to the original findings by Whittle et al. [8] we saw no significant loss (p = 0.5955) of regression during acute (Day 0) *P. falciparum* malaria. These assays were conducted on cryopreserved PBMCs but the magnitude of regressive capacity in acute (D0) *P. falciparum* infected children did not significantly differ from those detected using ex vivo assays on healthy control children (p = 0.2115, Figures 2F,G).

### Programmed Death-1 (PD-1) expression in *P. falciparum* infected and uninfected children

**Figure 3:**
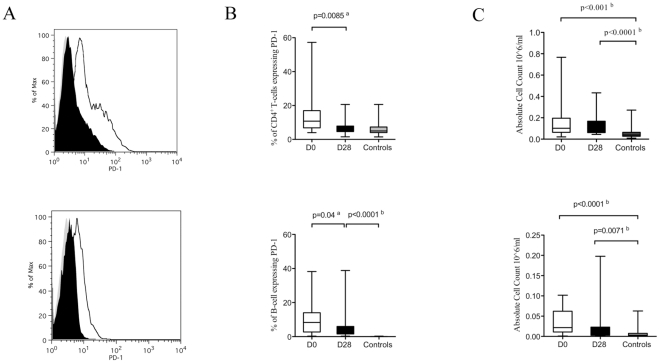
PD-1 expression is up-regulated on CD4 and CD19 positive cells during acute *P. falciparum* infection in children. A. Flow cytometry plots demonstrating increased expression of PD-1 on CD4^+^ T-cells (top row) and CD19 positive cells (bottom row) on Day 0 (black) compared to Day 28 (white). B. The percentage of programmed death-1 (PD-1) expressing, CD4 and CD19 positive cells in acute uncomplicated P. *falciparum* malaria infection (white box), on convalescence in the same donors (black box) and in age- and sex- matched healthy controls (grey box). C. The absolute number of programmed death-1 (PD-1) expressing, CD4 and CD19 positive cells in acute uncomplicated P. *falciparum* malaria infection (white box), on convalescence in the same donors (black box) and in age- and sex- matched healthy controls (grey box). All gates were set using appropriate isotype controls. ^a^ *P* values calculated using Wilcoxon rank comparison of matched pairs. ^b^ *p* values calculated using Mann-Whitney U statistical comparison of unmatched pairs.

During acute *P. falciparum* infection the percentage of PD-1 expressing CD19^+^ B-cells (p = 0.04) and CD4^+^ T-cells (p = 0.0085) was significantly higher than in convalescent samples (Figures 3A,B). This trend remains when results are expressed based on absolute numbers of CD19^+^ B-cells (Figure 3C). During acute *P. falciparum* malaria infection numbers of PD-1 expressing CD19^+^ B-cells (p = <0.0001) and CD4^+^ T-cells (p<0.001) were significantly higher than in healthy controls and during convalescence this number remained significantly higher than seen in the controls.

**Figure 4:**
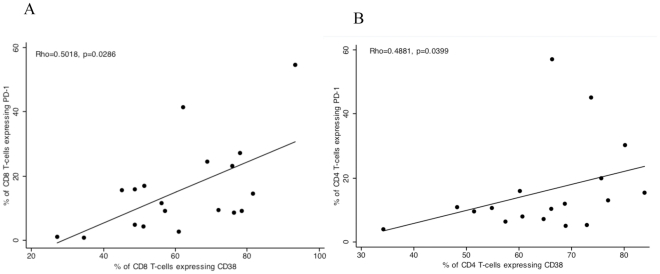
PD-1 expression correlates with CD38 expression during acute uncomplicated *P. falciparum* infection. PD-1 expression is shown on the y-axis and CD38 expression on the x- axis, each dot represents a single donor during acute (Day 0) uncomplicated P. *falciparum* malaria. There is a significant correlation between CD38 and PD-1 expression on CD8^+^ T-cells, A, and CD4^+^ T-cells, B.

Consistent with recent data [22], we found that during acute disease (D0) CD38 expression, a marker of activation, significantly correlated with PD-1 expression on CD8^+^ T-cells (Spearman's correlation coefficient Rho = 0.5018, p = 0.0286, Figure 4A) and also CD4^+^ T-cells (Spearman's correlations coefficient Rho = 0.4881, p = 0.0399), supporting the hypothesis that PD-1 expression is associated with cellular activation and is not purely a marker of functional exhaustion in these cells. However, this was not found to be the case for CD19^+^ cells (Spearman's correlation Rho = 0.4298, and p = 0.0663), data not shown.

## Discussion

This work directly assesses the effect of acute *P. falciparum* infection on the number and function of EBV-specific CD8^+^ T-cells ex vivo; in parallel with viral genome loads and traditional cell culture-based techniques measuring T-cell mediated regression of outgrowth of autologous B-cells. We show that the situation currently in The Gambia is different to that reported earlier [8], [14], now, children with acute uncomplicated *P.falciparum* malaria experience no elevation in EBV genome load in the blood compared to age- and sex-matched healthy controls, nor do they have any evidence of reduction in numbers or impairment of function of their EBV-specific CD8^+^ T-cells. We argue that these different findings could be due to a recent decline in chronic exposure to malaria in infancy and early childhood in the Gambia [18], [19].

Primary EBV infection, like Cytomegalovirus infection occurs in early childhood in the Gambia [23] and more recent data indicates that the age of EBV seroconversion continues to be low with 87% of children between 14 and 18mths of age found to be EBV seropositive in 2010 (S. Jayasooriya, unpublished data).

Loss of regression of EBV-infected B-cell outgrowth during acute malaria was first demonstrated in Gambian children over 25 years ago [8], at a time when the entomological inoculation rate in the country was several fold higher than the current day [24], [25]. Furthermore, in 2003, Njie et al. detected high EBV loads from PBMCs from Gambian children in the throes of acute malaria. Interestingly these did not fall in convalescent samples taken six weeks later but remained significantly higher than those detected in a cohort of healthy control children, albeit not matched for age- or sex in that report. In the present study, the EBV genome loads (using the same assay as Njie *et al.*) from malaria cases, both in acute and convalescent samples, are lower and not significantly different from those of age and sex-matched controls, although they remain significantly higher than the range typically seen for healthy virus carriers in the UK population [14]. Notably, there has been a considerable fall in malaria exposure in The Gambia from 2003 onwards [18], [19]. The likely difference in malaria exposure between the cohorts used by Whittle *et al.* and Njie *et al.* and the current study is highlighted by the dramatic change in entomological inoculation rates from Brefet, a rural community where part of the current study was based: 3.21 in 1991 to 0.62 in 2006 [24], [25]. It therefore seems possible that at the time of these earlier studies the life-long history of *P. falciparum* infection in childhood was still sufficiently chronic/recurrent as to render the EBV-host virus balance susceptible to modulation during a renewed bout of acute malaria. By contrast, given the recent decline in malaria incidence and increasing peak age of *P. falciparum* related hospital admissions in the Gambia [18], [19], it is plausible that most of our cases were experiencing a primary *P. falciparum* infection. We would argue that a single, primary *P. falciparum* infection is not sufficient to impair immunological control of EBV infection.

The work by Moormann et al., who studied two cohorts with differential malaria exposure in the Kisumu and Nandi areas of Western Kenya, is relevant here. They noted higher EBV genome loads in children, one to four years of age, living in areas holoendemic for malaria compared to those only sporadically exposed [12]. This is consistent with our data and supports the idea that chronic repeated exposure to malaria throughout a child's early years is required to alter the EBV-host balance, leading to high EBV loads and a greater likelihood of further increases linked to acute malarial episodes. To investigate this further would require following up children living in a holoendemic area from birth, to capture their first and subsequent malaria episodes, and to longitudinally examine EBV-specific immune responses and EBV viral loads.

Furthermore, we found that the function of EBV-specific CD8^+^ T-cells as measured by IFN-γ ELISPOT did not differ significantly between malaria cases, in acute stage or convalescence, and age- and sex- matched healthy controls. In a different study comparing children living under differing malaria exposure in Kenya, Moormann et al found that fewer children between five and nine years of age responded to pools of EBV peptides when coming from an area of high rather than sporadic malaria transmission [11]. The fact that bed net programmes target 0–5 year old children makes it conceivable that the most pronounced difference is detected in children above this age group. Of additional interest, despite a preservation of EBV-specific CD8^+^ T-cell responses, we find that PD-1, a marker reported to signify cellular exhaustion, is significantly up regulated in acute malaria and does not decline to control levels by day 28. However, PD-1 expression significantly correlates with CD38 expression on CD4^+^ and CD8^+^ T-cells which supports recent findings in HIV that PD-1 is not purely a marker of cell exhaustion [22] and reflects immune activation.

Our study has limitations that are worth discussion. Although previously reported data suggest a dramatic decline in malaria exposure in the country as a whole, our study design precludes exact knowledge of the exposure history of our study participants. However, we do know that 95% of three year old children followed up longitudinally in a birth cohort adjacent to our study's catchment area had no serological evidence of malaria exposure [18], and that a cohort study conducted in Brefet in the 2009 transmission season only identified one clinical case of malaria [26]. In addition to this we found only 24% of control children, mean age 7 years (range 1.5–16 years), were MSP-1(19) seropositive (data not shown). This is lower than previously quoted figures (30%, 0–7 years and 46%, 8–10 years) [27] Recent publications from the Gambia from differing peri-urban sites also confirm this decline [28], with one study demonstrating only a 4% seropositivity to MSP-1(19) in 0 to 14 years olds in 2009 [19].

Previous case-control studies have demonstrated that acute malaria infection can alter EBV host balance leading to an increase in EBV genome loads [15], [16], [17]. The cross-sectional cohort studies conducted by Moormann et al indicate that chronic malaria infection also plays a role in tipping the balance in favour of the virus [11], [12]. Our findings suggest that there is no impairment of EBV immunity during a primary acute *P. falciparum* infection and therefore suggest that alteration in EBV host balance is likely to be dependent on cumulative prior infections. A further longitudinal study of primary and cumulative acute *P. falciparum* infections and their impact on EBV host balance would be required to test this hypothesis.

Our findings, however, have more general implications adding to the mounting evidence that the decline in malaria seen in some parts of sub-Saharan Africa have wider implications than a reduction in morbidity and mortality from malaria alone. Additional changes have been seen in the rates of invasive bacterial diseases such as non-typhoidal salmonella in the Gambia [29], [30], and all cause mortality in children within regions where a reduction in malaria has been demonstrated [31]. Data to establish whether our observation translates into a reduced incidence of Burkitt's lymphoma are needed.

## Materials and Methods

### Ethics Statement

The Gambian Government/ MRC Laboratories Joint Ethics Committee approved this study. Participants were enrolled after individual written informed consent was obtained from the participant's parent/guardian.

### Donors

Thirty-two Gambian children (aged 1–15 years) enrolled into studies of severe and uncomplicated malaria were tested [20]. All presented with uncomplicated *P. falciparum* malaria, defined as a temperature >37.5°C within the last 48 hours, and ≥5000 parasites/µl detected by slide microscopy. Thirty-two age- and sex- matched healthy aparasitaemic controls were recruited from Brefet, a rural community where malaria transmission has dropped considerably [19]. All patients received standard care according to the Gambian Government Treatment Guidelines.

*P. falciparum* infected children were bled on admission (D0) and after 28 days (±3 days); controls were bled at one time point. 500 µl of blood was collected into an EDTA microcontainer for a full blood count (FBC) and DNA extraction. A further 4 mls was collected into heparinised vacutainers® (BD). PBMCs were separated on lymphoprep for use in ex vivo flow cytometric surface staining and IFN-γ ELISPOT assays.

### Viral load

EBV genome loads were assayed by quantitative real-time PCR, as described elsewhere [14], [32], [33]. The DNA extraction was performed on 200 µl whole blood using the Qiagen, QIAmp DNA Blood Mini kit. Results were expressed as EBV genome copies per 1×10^6^ PBMCs correcting for the PBMC percentage using the haematology count.

### Serology

IgG reactivity to EBV Viral Capsid Antigen (VCA) was measured by a commercial ELISA kit (DeMeditech, DEEBV01).

### HLA typing

Low resolution class I HLA typing was performed using sequence-specific primers (SSP) for the HLA-alleles of interest, A2, B35, B8 and B53, as previously described [34]. HLA-allele selection was determined by frequency in the Gambian population, determined by serological typing [35] and recently confirmed using high resolution sequence typing (Louis-Marie Yindom, unpublished data), and availability of known immunodominant EBV epitopes restricted through these class I HLA alleles (table 2). Based on polymorphisms in genes, some of which encode known CD8^+^ epitopes, two types of EBV can be distinguished. Epitopes used in this study have been defined based on work with type 1 EBV strains in Caucasians. While we did not sequence the resident strains of EBV in our donors, Njie et al. described previously that 90% of Gambian donors they studied carried type 1 EBV [14] indicating that the use of peptides derived from type 1 EBV is appropriate.

**Table 2:** EBV lytic and latent peptide pools.

EBV Protein	Peptide Pool	Epitope sequence	Epitope co-ordinates	HLA restriction
EBNA1	Latent	HPVGEADYF	407–415	B53
EBNA1	Latent	HPVGEADYFEY	407–417	B35
EBNA3A	Latent	QAKWRLQTL	158–166	B8
EBNA3A	Latent	YPLHEQHGM	458–466	B35
LMP2	Latent	FLYALALLL	356–364	A2
LMP2	Latent	CLGGLLTMV	426–434	A2
BRLF1	Lytic	YVLDHLIVV	109–117	A2
BZLF1	Lytic	EPLPQGQLTAY	54–63	B35
BMLF1	Lytic	GLCTLVAML	280–288	A2

**Note.** – EBNA, Epstein-Barr Nuclear Antigen. LMP, Latency Membrane Protein.

### ELISPOT assays

IFN-γ producing EBV specific lymphocytes were enumerated as previously described using the ELISPOT technique [36]. PBMCs were cultured in growth medium containing 10% human AB serum. 96-well plates were pre-coated with anti-IFN-γ mAb 1-DIK (MABTECH, Stockholm, Sweden). Ex vivo responses in growth medium, against EBV peptides (table 1), and PHA, both at a final concentration of 5 µg/ml) were screened at 10^5^ PBMCs per well in duplicate. Plates were incubated overnight at 37°C at 5% CO_2._ The cells were discarded the following day, and a biotinylated anti-IFN-γ mAb, 7-B6-1 biotin (MABTECH), was added at 1 µg/ml for 3 hours at room temperature, followed by streptavidin-conjugated alkaline phosphatase (MABTECH) for 2 hours. Cytokine-producing cells were detected after a 30-min reaction with 5-bromo-4-chloro-3-indolyl phosphate and nitro blue tetrazoliym using an alkaline phosphatase-conjugate substrate kit (Bio-Rad, Richmond, CA). Spots were counted using an automated AID ELISPOT plate reader©. PBMCs were screened for reactivity to EBV using pools of commonly recognised CD8^+^ lytic and latent peptides as shown in table 1 (obtained from the Weatherall Institute for Molecular Medicine (WIMM), Oxford). Negative control background values were subtracted from all responses. Negative values were set to zero and classified as non-responders.

### Cell Surface and Tetramer Flow Cytometric Staining

Ex vivo, 1×10^6^ PBMCs per MHC Class I tetramer (for lower cell counts all four tetramers were pooled) were stained for 1 hour at 4°C in the dark. MHC class I Tetramers containing GLCTLVAML (A2_GLC), CLGGLLTMV (A2_CLG), EPLPQGQLTAY (B35_ EPL) and RAKFKQLL (B8_RAK) peptides were used, conjugated to Phycoerythrin, PE, (A2_GLC synthesised at the Institute for Cancer Studies, University of Birmingham, the others from the WIMM). Tetramer staining was followed by surface marker staining (30 min, 4°C in the dark) according to the manufacturer's protocol (BD Biosystems), using the following antibodies: Programmed Death-1 (PD-1) Fluorescein Isothiocyanate (FITC), CD4 PerCP, CD19 PE-Cy7, CD38 Allophycocyanin (APC), (BD Biosystems) CD8 Pacific Blue, CD27 APC-Alexa750, (Ebioscience) CD28 ECD (Beckman Coulter) and CD3 cascade yellow (Dako). Appropriate isotype control antibodies, IgG1 FITC, IgG1 PerCP, IgG1 PE-Cy7, IgG1 APC, (BD Biosystems) IgG2a Pacific Blue, IgG1 APC-Alexa750, (Ebioscience) and IgG1 Cascade Yellow (Dako), were used. Samples were acquired on a Cyan™ ADP flow cytometer using Summit software (Beckman Coulter). Analysis was performed using FlowJo (Tree Star Inc.) and isotype controls were used to set the gating.

### Regression Assays

These were carried out on cryopreserved PBMCs in the following malaria season. PBMCs from uncomplicated and complicated *P. falciparum* malaria cases recruited from the same study platform [20] were used. In addition, we performed ex vivo regression assays on healthy control children, recruited from Brefet during the same year. Assays were performed as previously described [9] except cells were seeded into U-bottom microtest plates in doubling dilutions from 4×10^4^ to 1.2×10^3^ cells/well. The strength of regression was expressed as the initial cell seeding necessary to give a 50% incidence of regression among replicate wells as calculated using the Reed-Muench formula.

### Statistics

Viral genome load, serology, ELISPOT and flow cytometry data were plotted and statistically analysed using GraphPad Prism software (Version 5; GraphPad). Wilcoxon rank and Mann-Whitney U non-parametric tests were used for comparisons of matched and unmatched data respectively. STATA (version 11) was used to calculate Spearman's rank correlation coefficients.
